# Diseases of Digestion, Urinary and Genitive Organs

**Published:** 1887-01

**Authors:** 


**Diseases of Digestion, Urinary, and Generative Organs.** Illustrated by 106 fine wood engravings. Being Volume II. of the Handbook of Practical Medicine. By Dr. Hermann Eichhorst, Professor of Special Pathology and Therapeutics, and Director of the University Medical Clinic in Zurich. This is Vol. VI. of Wood's Library for 1886. New York: William Wood & Company.

Our subscribers who are not this year taking " Wood's Library " are losing some excellent books, indeed we think the series for 1886, in some respects, the best of any year. All of the volumes on " Practical Medicine," by Dr. Hermann Eichhorst, has been worthy of their title. They are eminently practical and thoroughly up to the present knowledge.

